# Silibinin Induced Human Glioblastoma Cell Apoptosis Concomitant with Autophagy through Simultaneous Inhibition of mTOR and YAP

**DOI:** 10.1155/2018/6165192

**Published:** 2018-03-26

**Authors:** Zhuan-Li Bai, Vincent Tay, Shu-Zhong Guo, Juan Ren, Mao-Guo Shu

**Affiliations:** ^1^Department of Plastic, Aesthetic and Maxillofacial Surgery, First Affiliated Hospital of Xi'an Jiaotong University, Xi'an, Shaanxi, China; ^2^Department of Plastic, Reconstructive and Aesthetic Surgery, Singapore General Hospital, Singapore; ^3^Department of Radiotherapy, First Affiliated Hospital of Xi'an Jiaotong University, Xi'an, Shaanxi, China

## Abstract

Silibinin, also known as silybin, is the major flavonolignan isolated from* Silybum marianum*. Although previous reports demonstrated that silibinin exhibits significant tumor suppressor activities in various cancers by promoting cell apoptosis, it was also shown to trigger autophagy to counteract apoptosis induced by exogenous stresses in several types of cells. However, there is no report to address the role of silibinin induced autophagy in human A172 and SR glioblastoma cells. Our study showed that silibinin treatment not only inhibited the metabolic activities of glioblastoma cells but also promoted their apoptosis through the regulation of caspase 3 and PARP-1 in concentration- and time-dependent manners. Meanwhile, silibinin induced autophagy through upregulation of microtubule-associated protein a light chain 3- (LC3-) II. And autophagy inhibition with chloroquine, a lysosomotropic agent, significantly enhanced silibinin induced glioblastoma cell apoptosis. Moreover, silibinin dose-dependently downregulated the phosphorylation levels of mTOR at Ser-2448, p70S6K at Thr-389, and 4E-BP1 at Thr-37/46. Furthermore, the expression of YAP, the downstream effector of Hippo signal pathway, was also suppressed by silibinin. These results suggested that silibinin induced glioblastoma cell apoptosis concomitant with autophagy which might be due to simultaneous inhibition of mTOR and YAP and silibinin induced autophagy exerted a protective role against cell apoptosis in both A172 and SR cells.

## 1. Introduction

Glioblastoma multiforme (GBM) is the most common and aggressive primary malignant brain tumor, which constitutes 16% of all primary central nervous system neoplasms. And its age-adjusted incidence rate is about 3.2 per 100,000 populations [1–3]. Unfortunately, the therapeutic response and prognosis of GBM are very poor, and current treatment regimens combining surgical resection, radiation, and chemotherapy only lead to an increase in median overall survival from 12.1 to 14.6 months [4]. Therefore, exploring novel therapeutic compounds is of extreme importance to improve survival for GBM patients.

**Figure 1:**
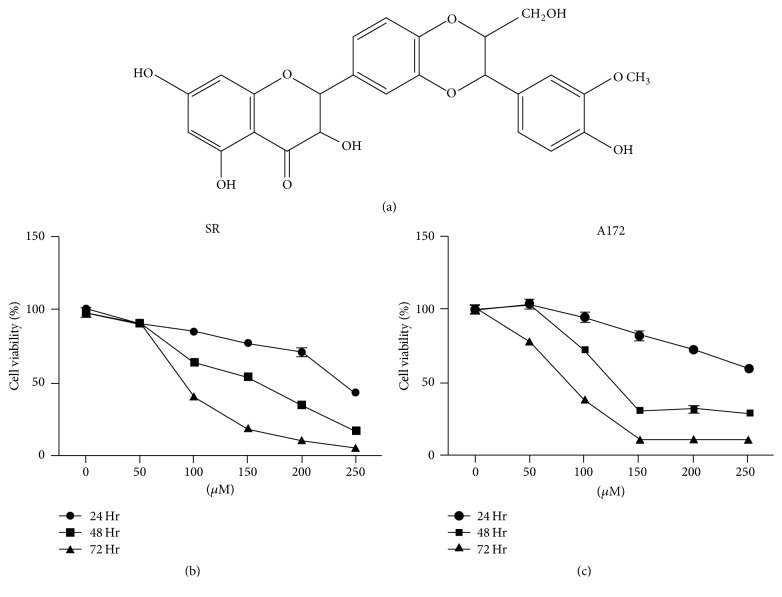
Silibinin inhibited cellular viability in glioblastoma cells. (a) Chemical structure of silibinin, a major flavonolignan isolated from the seeds of milk thistle. ((b) and (c)) Concentration- and time-dependent inhibition of cellular viability of silibinin on SR and A172 cells as measured by MTT assay. Data are presented as mean ± SD (*n* = 3 in each group).

Silibinin, the major flavonolignan also known as silybin (Figure 1(a)) isolated from* Silybum marianum* (L.) Gaertn, has been used as an antioxidant and hepatoprotective agent [5, 6]. Studies have shown that silibinin exhibits a multitude of pharmacological effects in hepatobiliary disorders, including hepatitis and cirrhosis [7, 8]. And silibinin is found in dietary supplements and had been used as an agent against alcoholic liver disease and Child's type A liver cirrhosis in Europe, Asia, and the United States in recent years [9, 10]. In addition, silibinin was reported to have significant tumor suppressor functions in various cancers, including cancers of the breast, prostate, lung, bladder, colon, skin, and kidney [11–20]. It can significantly suppress the invasion and metastasis of cancer cells [21, 22]. Specifically, Momeny and colleagues demonstrated that silibinin significantly suppressed metabolic activity and cell proliferation in human glioblastoma U87 MG cells [23]. Moreover, silibinin enhances the sensitivity of various human glioblastoma cell lines to several chemotherapeutic drugs including temozolomide, etoposide, and irinotecan [24]. Also silibinin was shown to be involved in regulating autophagy of glioblastoma cells [25]. Therefore, silibinin has the potential to be a useful therapeutic drug for glioblastoma [26]. However, the exact molecular mechanisms responsible for the antitumor effects of silibinin on glioblastoma cells are yet to be fully elucidated. The aim of this study is to investigate the effects of silibinin on the growth, apoptosis, and autophagy of human glioblastoma cells.

## 2. Materials and Methods

### 2.1. Cell Line and Silibinin Treatment

The human glioblastoma cell lines, A172 and SR cells, were grown as a monolayer in RPMI 1640 medium (Invitrogen, Auckland, New Zealand) supplemented with 10% fetal bovine serum (Invitrogen) in 5% CO_2_ at 37°C. The cultures were then treated with 0, 50, 100, 150, 200, and 250 *μ*M of silibinin (Sigma, St. Louis, Missouri, USA), respectively, for different time duration.

### 2.2. Microculture Tetrazolium Assay

A microculture tetrazolium assay was performed to determine the inhibitory effect of silibinin on the metabolic activity of A172 and SR cells. The cells were plated onto 96-well plates at a density of 5000 cells/well. After incubation at 37°C for 24 h, the cells were exposed to silibinin at 0, 50, 100, 150, 200, and 250 *μ*M for different time duration (i.e., 24, 48, and 72 h). 3-(4,5-Dimethylthiazol-2-yl)-2,5-diphenyltetrazolium bromide (MTT) solution (0.5 mg/mL) of 200 *μ*L was added to each well and the cells were further incubated at 37°C for 4 h. After dissolving the precipitated formazan with 100 *μ*L of dimethyl sulfoxide (DMSO), the optical densitometry was measured at the wavelength of 490 nm. The inhibition rate of silibinin was evaluated using the following equation: inhibition rate (%) = (1  −  OD_exp_/OD_con_) × 100, where OD_exp_ and OD_con_ are the optical densities of treated and untreated cells, respectively.

### 2.3. Flow Cytometry for Determining Apoptotic Population

To evaluate the apoptosis-inducing effects of silibinin in both A172 and SR cells, we follow the method of Chakrabarti and Ray (2015) [27]. Briefly, the glioblastoma cells were exposed to different concentrations of silibinin for 48 h and then collected by centrifugation at 2,000*g* for 6 min at room temperature. The cells were washed twice with ice-cold PBS and resuspended in 1x binding buffer (BD Biosciences, San Jose, CA, USA) at a concentration of 1 × 10^6^ cells/mL. Then, 100 *μ*L of each cell resuspension was transferred to a 5 mL FACS analysis tube and 5 *μ*L of Annexin V-fluorescein isothiocyanate (FITC) and 5 *μ*L of propidium iodide (PI) solution (BD Biosciences) were added to each tube. According to the manufacturer's protocol, cells were incubated for 15 min at room temperature in the dark after brief and gentle vortexing. Finally, 400 *μ*L 1x binding buffer was added to each tube and flow cytometric analysis was performed to detect apoptotic cells.

### 2.4. Confocal Fluorescence Microscopy

A172 and SR cells were cultured on slides and transiently transfected with mRFP-GFP-LC3 adenovirus. Then the cells were exposed to different concentrations of silibinin, combined with or without 10 *μ*M chloroquine (CQ) for 24 h. After being washed with PBS for three times, the slides were blocked with glycerol and LC3 puncta were visualized with a confocal fluorescence microscope (Leica TCS SP5 II).

### 2.5. Western Blot Analysis

Western blot was carried out according to the method previously reported by Chakrabarti and Ray (2015) with minor modification [27]. Both A172 and SR cells were lysed in the lysis buffer (50 mM Tris-HCl, pH7.4, 1 mM PMSF, and 5 mM EGTA) and homogenized by sonication after exposure to different concentrations of silibinin for 48 h. Then the whole cell lysates were centrifuged at 16,000*g* at 4°C for 30 min and the supernatants were used to prepare protein samples. Protein concentrations of the samples were quantified by the modified Bradford method after staining with Coomassie Plus protein reagent (Pierce Biotechnology, Rockford, IL). Protein samples (30 *μ*g) were resolved by 4–20% gradient SDS-PAGE and transferred to the polyvinylidene fluoride (PVDF) membranes (Millipore, Billerica, MA). After blocking in 5% nonfat milk for 1 h, PVDF blots containing the resolved proteins were subsequently probed with a primary antibody (1 : 1000) against PARP, cleaved PARP, caspase 3, *β*-actin, LC-3 I/II, P62, mTOR, p-mTOR (Ser2448), YAP, p-S6k (Thr389), and p-4E-BP1 (Thr37/46) (Cell signaling Technology, Danvers, MA) overnight at 4°C. After washing with TBS for five times, the blots were further incubated with horseradish peroxidase conjugated goat anti-mouse or anti-rabbit IgG antibody (Santa Cruz Biotechnology, Santa Cruz, CA) at 1 : 3000 dilution for 60 min to detect the primary antibody. Then, blots were thoroughly washed with TBS for five times and incubated with ECL reagents and exposed to X-OMATAR films for autoradiography. Autoradiograms were scanned using Canon CanoScan 9000F Mark II Photo Scanner.

### 2.6. Statistical Analysis

Data are expressed as mean ± standard deviation. All experiments were performed in triplicate. For statistical analysis, Student's *t*-test and one-way analysis of variance were applied. To compare the control group with the experimental groups, Dunnett's multiple comparison test was used. *P* values less than 0.05 were considered statistically significant.

## 3. Results

### 3.1. Silibinin Inhibited the Metabolic Activity of Glioblastoma Cells

It has been demonstrated that exposure to silibinin alone for 24 h has limited impacts on cellular viability in both glioblastoma cells and glioblastoma stem cells [27]. However, whether prolonging the exposure time could enhance its inhibitory effects has not been reported. In this study, the effect of silibinin on the cellular viability of glioblastoma cell lines, SR and A172, was evaluated using the MTT assay at different concentrations of silibinin (50, 100, 150, 200, and 250 *μ*M) and treatment durations (24, 48, and 72 h). A concentration- and time-dependent inhibition of SR and A172 cellular metabolic activity was demonstrated as shown in Figures 1(b) and 1(c). After treatment with 150 *μ*M silibinin for 72 h, more than 70% of the glioblastoma cells lost their viability.

### 3.2. Silibinin Induced Glioblastoma Cell Apoptosis via Cleavage of Caspase 3 and PARP

**Figure 2:**
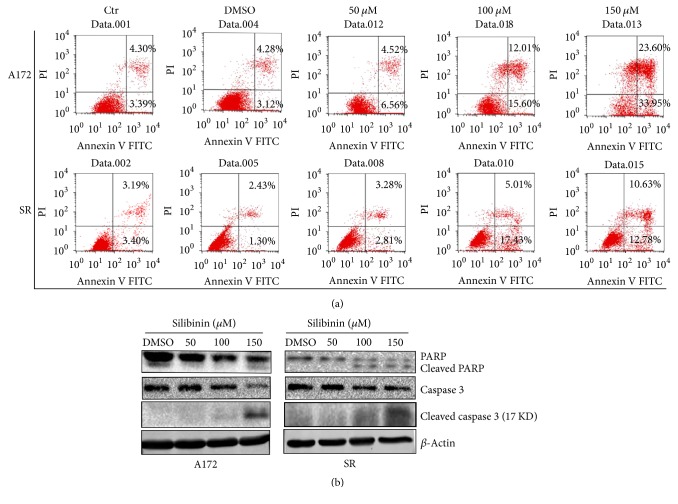
Silibinin induced apoptosis in glioblastoma cells. (a) Glioblastoma cells were treated with different concentrations of silibinin for 48 h; then the apoptotic index was calculated after flow cytometric analysis. (b) After treatment with different concentrations of silibinin for 48 h, total lysates of A172 and SR cells were analyzed for caspase 3, cleaved caspase 3, and PARP cleavage by western blotting. *β*-Actin was used as a loading control. Data are presented as mean ± SD (*n* = 3 in each group).

To confirm that apoptosis was involved in silibinin induced decrease in glioblastoma cell viability, SR and A172 cells were exposed to different concentrations of silibinin for 48 h and then subjected to flow cytometric analysis after Annexin V FITC/PI double staining. The results showed that the percentage of Annexin V-positive apoptotic cells increased with the concentration of silibinin, whereas apoptosis was seldom observed in DMSO-treated and the control cells (Figure 2(a)).

Next, the proapoptotic effect of silibinin was further confirmed by cleavage analysis of both caspase 3 and poly (ADO-ribose) polymerase 1 (PARP-1). Upon apoptosis, caspase 3, the critical apoptosis executioner, is activated by proteolytic processing of its inactive zymogen into activated p17 and p12 fragments and is responsible for cleavage of a large variety of proteins including PARP-1. Our results showed that silibinin treatment led to the cleavage of caspase 3 and PARP-1 in both SR and A172 cells in a concentration-dependent manner (Figure 2(b)), indicating that silibinin induces glioblastoma cell apoptosis via a caspase-dependent PARP-1 cleavage, which has been widely considered to be a hallmark of apoptosis.

### 3.3. Silibinin Induced Autophagy in Glioblastoma Cells

**Figure 3:**
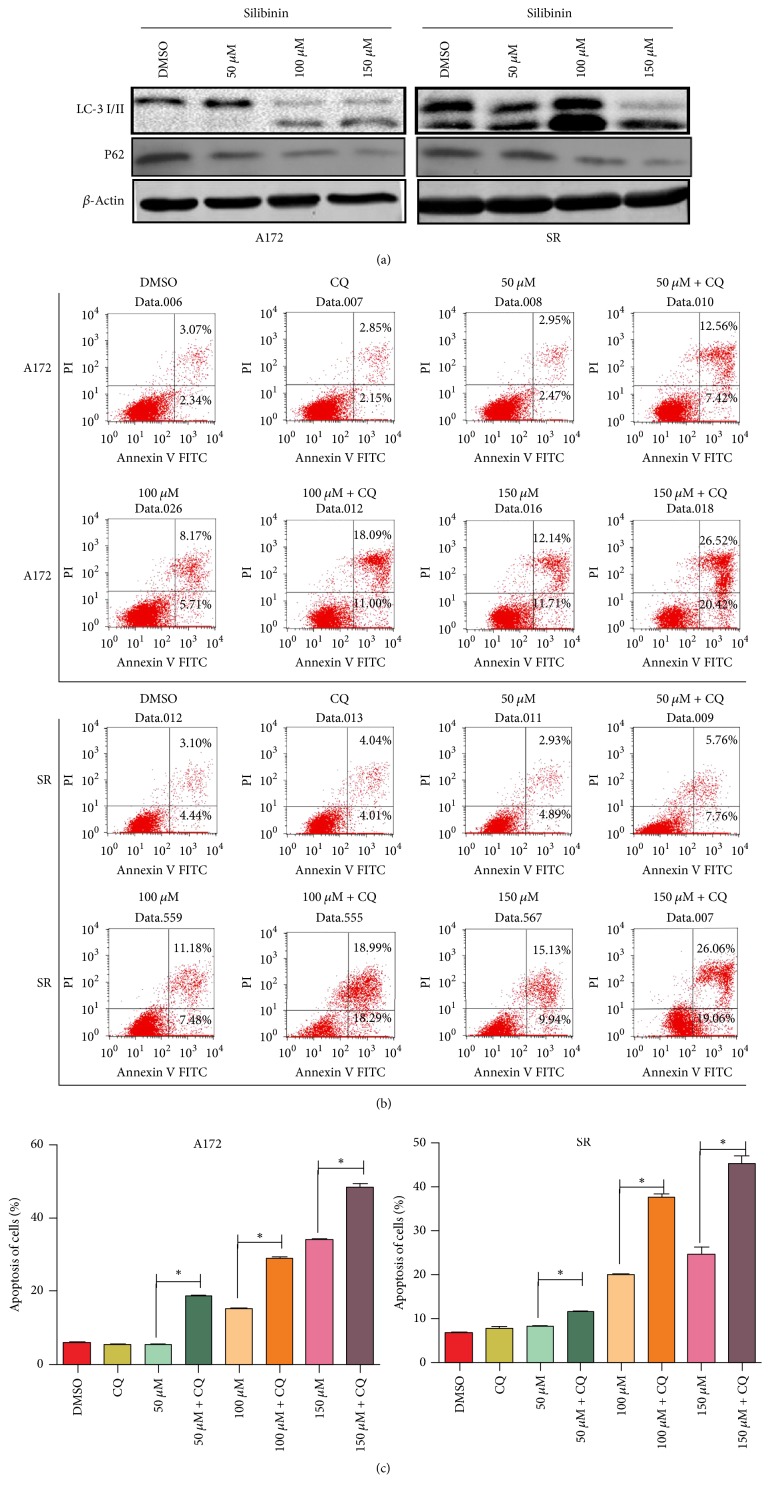
Silibinin induced protective autophagy in glioblastoma cells. (a) Expression levels of LC3-II and P62 were detected by western blotting. Both A172 and SR cells were treated with different concentrations of silibinin for 48 h. *β*-Actin was used as a loading control. (b) Glioblastoma cells were treated with different concentrations of silibinin, combined with or without chloroquine (CQ) for 48 h; then the apoptotic index was calculated after flow cytometric analysis. (c) Apoptosis quantitative data of glioblastoma cells. Data are presented as mean ± SD (*n* = 3 in each group). ^*∗*^*P* < 0.05 versus the control group.

**Figure 4:**
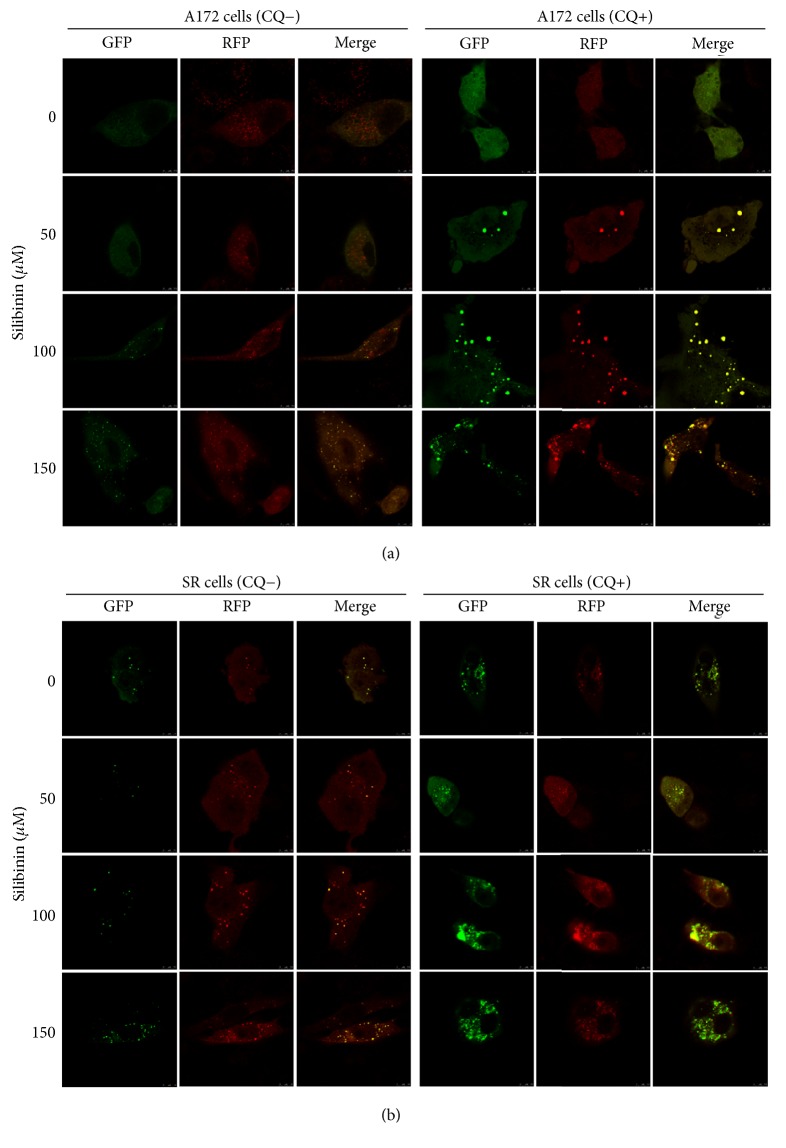
Silibinin enhances autophagic flux in glioblastoma cells. A172 (a) and SR (b) cells were transfected with mRFP-GFP-LC3 adenovirus and treated with different concentrations of silibinin, combined with or without CQ for 24 h. Green, red, and yellow puncta were visualized with Leica TCS SP5 II confocal fluorescence microscope.

Previous reports demonstrated that silibinin can induce autophagy in addition to apoptosis in fibroblast and several types of tumor cells. However, whether silibinin could induce autophagy in glioblastoma cell has not been reported. During mammalian autophagy process, one of the hallmark events is the conversion of LC3-I to LC3-II via proteolytic cleavage and lipidation, which is then covalently modified and localized to autophagosomes. In this study, we found that silibinin treatment promoted the conversion of LC3-I to LC3-II and the degradation of P62 in a concentration-dependent manner in both A172 and SR cells (Figure 3(a)), which represents an enhanced autophagic flux, whereas the solvent DMSO alone showed no effects. The result of mRFP-GFP-LC3 adenovirus transfection assay further confirmed that silibinin could dose-dependently enhance autophagic flux in both cells (Figure 4).

### 3.4. Chloroquine Enhanced Silibinin Induced Glioblastoma Cell Apoptosis

In view of the fact that autophagy has been considered as a double-edged sword and functions as both tumor suppressor and tumor promoter, we further evaluated the effect of autophagy inhibition on silibinin induced glioblastoma cell apoptosis. Chloroquine, a lysosomotropic agent, which can prevent endosomal acidification and lead to inhibition of both fusion of autophagosome with lysosome and lysosomal protein degradation, has been well-documented to be an autophagy inhibitor. Although chloroquine alone showed no obvious effects on glioblastoma cell apoptosis, it significantly augmented the apoptosis induced by different concentrations of silibinin (Figure 3(b)). This finding suggested that chloroquine augmented the proapoptotic effect of silibinin on glioblastoma cells and/or where autophagy may play a protective role in apoptosis induced by silibinin.

### 3.5. Silibinin Inhibited the Phosphorylation of mTOR and Its Downstream Effectors

Akt/mTOR signaling pathway has been well-known to play critical roles in controlling cell proliferation and apoptosis as well as autophagy induction through modulation of protein translation via phosphorylation of p70 ribosomal protein S6 kinase (p70 S6K) and eukaryotic initiation factor 4E binding protein 1 (4E-BP1). And its deregulation is commonly found in GBM. Therefore, we determined the effects of silibinin on the phosphorylation levels of mTOR, p70S6K, and 4E-BP1. Our results showed that silibinin inhibited phosphorylation of mTOR at Ser-2448, p70S6K at Thr-389, and 4E-BP1 at Thr-37/46 in a concentration-dependent manner in both A172 and SR cells.

### 3.6. Silibinin Downregulated the Protein Level of YAP

**Figure 5:**
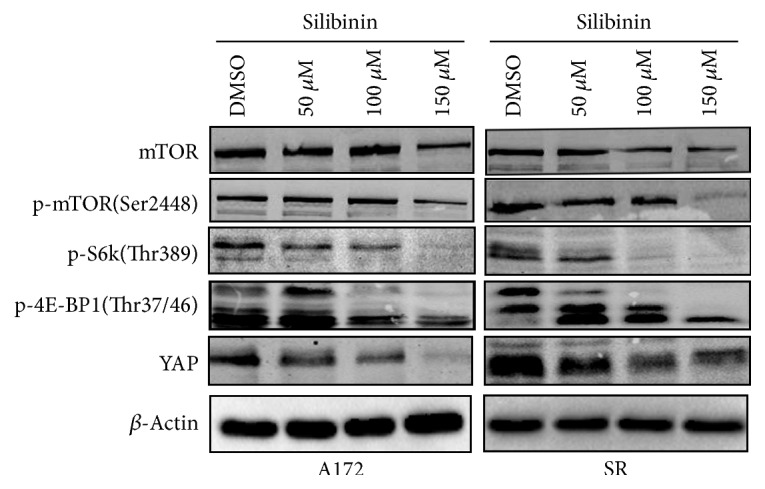
Silibinin inhibited mTOR signal pathway and downregulated YAP expression. Both A172 and SR cells were treated with different concentrations of silibinin for 48 h, and then levels of total mTOR, phosphorylated mTOR, phosphorylated S6K, phosphorylated 4E-BP1, and YAP were determined by western blotting. *β*-Actin was used as a loading control.

Besides Akt/mTOR signaling cascade, the Hippo pathway is also a master regulator of organ size and is often involved in tumorigenesis. It has been reported that the protein level of YAP, one of the downstream transcriptional coactivators of Hippo signaling pathway, was tightly correlated with mTOR activity in several types of tumor cells, including pulmonary lymphangioleiomyomatosis, hepatic angiomyolipomas, and renal angiomyolipomas [28]. To test whether silibinin induced downregulation of YAP, A172 and SR cells were exposed to silibinin for 48 h. As a result, the protein level of YAP decreased significantly in response to the increase of the concentration of silibinin (Figure 5). And YAP expression levels seemed to be correlated with the phosphorylation levels of mTOR, p70S6K, and 4E-BP1, implying that mTOR signaling pathway might be an important regulator in controlling YAP expression in glioblastoma cells.

## 4. Discussion

In this study, we found that silibinin induced concentration-dependent apoptosis and autophagy in glioblastoma cells. The apoptosis induced by silibinin was demonstrated to have resulted from caspase-dependent PARP cleavage, while the induced autophagy was inferred from the increase in conversion of LC3-I to LC3-II and the decrease of P62 protein level. Interestingly, we noted that cotreatment with chloroquine, an inhibitor of autophagy, significantly augmented the proapoptotic effect of silibinin. This finding may imply that either chloroquine enhanced the induction of apoptosis by silibinin or autophagy induced by silibinin may have rescued some cell population from apoptosis. Even though chloroquine had been shown to induce apoptosis via the p53 and TRAIL pathways at 30 *μ*M and higher by other authors [29, 30], our control experiment with 10 *μ*M of chloroquine did not induce apoptosis in SR or A172 cells. Based on emerging literature that has suggested the role of autophagy in the survival of cancers cells [31–35], the role of silibinin in impeding apoptosis due to increased autophagy in some cell population is more plausible.

Autophagy is an intrinsic intracellular biological activity to recycle intracellular organelles and macromolecules to maintain cellular homeostasis [36, 37]. During autophagy, damaged or useless organelles, proteins, and portions of the cytoplasm are sequestered in a double membrane structure called autophagosome, which is subsequently delivered to the lysosome for degradation [38]. In recent years, autophagy has received much attention for it can be either prosurvival or prodeath depending cellular context [39]. In the scenario of cancer cells, a large body of reports proved that autophagy can promote cell survival through avoiding accumulation of deleterious organelles and proteins that has resulted from insults of the bodily immune response or chemotherapeutics [32, 40, 41]. Davids et al. had shown that induction of autophagy could enhance the protective effect of hypericin against the apoptosis of melanoma cells induced by Ultraviolet A [42]. Jiang et al. also demonstrated that silibinin inhibited the expression of p53, which facilitated NF-*κ*B activation and mediated autophagy in a positive feedback loop. The induced autophagy was noted to have a cytoprotective effect against mitomycin C-induced apoptosis which can be abolished with inhibition of autophagy [43, 44]. Therefore, our finding suggested that the proapoptotic effect of silibinin on glioblastoma cells may be partially impeded by autophagy which is consistent with existing literature for other cell lines. However, more research will be required to elucidate the mechanism involved in the upregulation of autophagy. It may be solely due to a mechanistic effect of silibinin on NF-*κ*B activation and/or due to accumulating damaged organelles or protein from the insult of silibinin treatment.

Previous reports have demonstrated that silibinin inhibits mTOR pathway in both renal cell carcinoma and multiple myeloma cells [45–47]. Here, we showed that silibinin inhibited the phosphorylation of mTOR, p70S6K, and 4E-BP1 in human glioblastoma cells. Moreover, we found that silibinin treatment induced a concentration-dependent downregulation of YAP, the downstream effector of Hippo pathway, which had never been reported before. Since both the mTOR pathway and the Hippo pathway are well-known to play critical roles in controlling cell proliferation and apoptosis, further research is needed to discern their respective roles in silibinin induced apoptosis.

In conclusion, our studies demonstrated that silibinin suppressed YAP expression in addition to inhibition of mTOR pathway in glioblastoma cells. Moreover, silibinin treatment concomitantly induced apoptosis and autophagy, and chloroquine strengthened the proapoptotic effects of silibinin. Therefore, we suggest that autophagy inhibitors might be included in the multidrug treatment regimen for glioblastoma.
